# The role of negative life events and parental mental health in adolescent self-regulation: insights from the longitudinal ABCD study

**DOI:** 10.1186/s13034-025-00991-5

**Published:** 2025-12-01

**Authors:** Emely Reyentanz, Yulia Golub, Mandy Roheger, Mira Vasileva

**Affiliations:** 1 Department of Child and Adolescent Psychiatry, Psychosomatic and Psychotherapy, School of Medicine and Health Sciences, Carl von Ossietzky Universität Oldenburg, Oldenburg, Germany; 2 Ambulatory Assessment in Psychology, Department of Psychology, Carl von Ossietzky Universität Oldenburg, Oldenburg, Germany

**Keywords:** Self-regulation, Adolescents, Negative life events, Parental mental health

## Abstract

**Background:**

Adolescence is a critical period for the development of mental health problems, with self-regulation playing a crucial role as a protective factor. However, little is known about the self-regulation development in adolescence and how this is influenced by environmental factors such as negative life events (NLEs) and parental mental health problems. This study aimed to examine changes in self-regulation and the predictive effect of NLEs and parental mental health problems on self-regulation.

**Methods:**

We included a sample of *N* = 2803 adolescents from the ABCD study. We explored changes in self-regulation by comparing variables between the ages of 11–12 and 13–14 (behavioral and cognitive self-regulation) and the ages of 12–13 and 13–14 (emotional self-regulation). We also compared self-regulation changes in adolescents with and without a history of NLEs and with and without parents with clinically significant mental health problems. Using linear regression, we analyzed the predictive effect of NLEs and parental mental health problems on self-regulation two years later.

**Results:**

Adolescents showed a small increase in mean cognitive self-regulation (η^2^_part_ = 0.22) and expressive suppression as part of emotional self-regulation (η^2^_part_ = 0.07), and a small decrease in behavioral self-regulation (η^2^_part_ = 0.09). The results of the regression analysis indicate small, significant effects of NLEs and parental mental health problems on adolescent self-regulation. Self-regulation at the first assessment significantly predicted later self-regulation.

**Conclusions:**

Our results suggest that self-regulation still develops in early adolescence, marked both by improvements in some components of self-regulation and difficulties in others. To better understand developmental trajectories and determinants of self-regulation, prospective longitudinal studies starting earlier in development and covering a longer period are needed.

**Supplementary Information:**

The online version contains supplementary material available at 10.1186/s13034-025-00991-5.

## Introduction

Self-regulation involves internal processes of regulating emotions, behavior, and thoughts to adapt to a changing context or achieve goals [1, 2]. It encompasses emotional (e.g. strategies such as cognitive reappraisal or expressive suppression of emotions), behavioral (e.g. impulse control) and cognitive (e.g. effortful control) components [1, 2]. One major challenge in previous research on self-regulation hampering direct comparison and replication has been the inconsistent operationalization and varying definitions of the construct and its components. For instance, terms such as executive functioning and effortful control are often used interchangeably, although they are seen as components of self-regulation [1]. Furthermore, previous studies have often only focused on single components of self-regulation. Clinical research, for example, often investigates emotional self-regulation and related therapeutic approaches to improve emotion regulation skills in order to reduce mental health problems [3, 4]. In other studies, self-regulation has been examined as part of other constructs such as temperament [5] or even personality traits [6] that theoretically are expected to show less development over time. In contrast, the current study examines self-regulation as a multi-component construct - encompassing emotional, behavioral, and cognitive aspects - that develops over time.

The development of self-regulation skills is assumed to happen in consecutive stages [7]. To date, its development has been studied primarily in infancy [8] and throughout (early) childhood [9], when the first patterns of self-regulation skills, such as the modulation of attention [10], emerge. Early childhood and preschool age (up to the age of approx. five years) are considered a sensitive period for brain maturation and thus for rapid developmental changes in self-regulation [9, 11]. In this time, executive functions, such as inhibition or working memory, significantly develop [12]. These skills are, in turn, the basis for the regulation of emotions, behavior and cognitions [1, 9]. Nonetheless, adolescence is also an important period for brain maturation [13] during which frontal brain regions involved in the regulation of emotions, behavior, and attention develop [2, 14, 15]. Single findings also indicate changes in behavioral components of self-regulation [16], although the results on the extent and direction of these changes are inconsistent [17, 18].

**Fig. 1:**
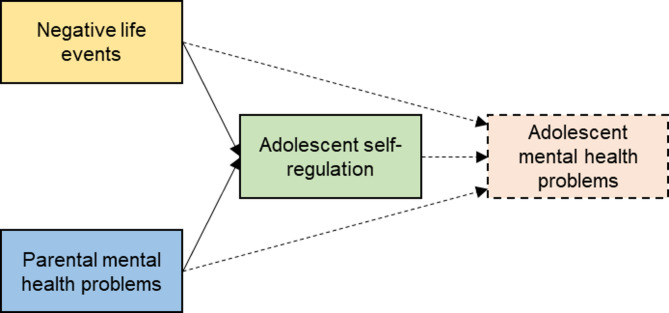
Assumed theoretical model of the association between negative life events, parental mental health problems and adolescent self-regulation (based on e.g. Dvir et al., 2014; Gould et al., 2012; Karl et al., 2024; King et al., 2013; Lackner et al., 2018; Lin et al., 2024). Dashed lines indicate associations that are not examined in this study due to low internal consistency of the measures of adolescent mental health problems.

Important determinants of self-regulation include environmental influences such as NLEs and characteristics of caregiving [2] (see Fig. 1). Previous studies have linked the experience of NLEs - defined here as uncontrollable adverse events [19] such as death or serious injury of close relatives or financial problems of the family - to lower self-regulation skills [17, 20–22]. Neurobiological studies show that NLEs can alter brain structure and function (e.g. cortico-limbic resting-state functional connectivity), which may underlie these self-regulatory problems. NLEs also may impact self-regulation changes in adolescence: King, Lengua [17] reported that effortful control improved faster over three years in adolescents with more NLEs than in participants with fewer NLEs, suggesting possible compensatory effects.

Parental mental health problems also play a key role in shaping self-regulation development [23, 24]. Brain changes in relation to parental mental health problems were reported [25], indicating neurobiological changes such as in brain network connectivity between various regions as possible mediating mechanisms. Morris, Silk [26] postulated a tripartite model of the parental influence on child emotion regulation through observational learning, parenting practices, and the emotional climate in the family. While there is extensive research on the link between parental mental health and child mental health problems [27] and emotional regulation [23] in children and adolescents, less is known about its impact on cognitive and behavioral self-regulation. Ruof, Elam [28] reported associations between maternal mental health problems and adolescent effortful control, and data from the Adolescent Brain Cognitive Development (ABCD) Study^®^ [29] indicate a small effect of parental mental health problems at baseline (child age 9–10 years) on child impulsivity 2 years later [30].

Given the role of self-regulation as a transdiagnostic factor for mental health problems [31, 32], and the findings that adolescence is the peak age for the onset of psychiatric disorders (e.g. disorders due to substance use or addictive behavior) [33], a better understanding of the effect of environmental determinants such as NLEs and parental mental health problems on self-regulation and its developmental changes during adolescence could provide important insights into the mechanisms underlying the development of child mental health problems. To date, the majority of research has focused on investigating single components of self-regulation, such as emotional self-regulation, which was shown to mediate the association between NLEs and psychiatric disorders in children and adolescents [34, 35] and between parental and child mental health problems [24]. However, since several findings suggest that all three components of self-regulation play a crucial role in the development of mental health problems [36–38], the aim of this study was to investigate them in parallel with the same sample of adolescents. These findings could provide opportunities for more specific preventive and therapeutic approaches to improve self-regulation skills for children and adolescents who experienced NLEs or have parents with mental health problems. Therefore, this study pursues the following objectives:

1. Investigation of individual and mean changes in self-regulation in adolescents aged 11–12 and 13–14 years.
2. Investigation of differences in mean changes in self-regulation in adolescents aged 11–12 and 13–14 years.
  - between adolescents with and without experience of NLEs.
  - between adolescents with and without parents with clinically significant mental health problems.
3. Examining the predictive effect of NLEs and parental mental health problems at the age of 11–12 years on adolescent self-regulation at 13–14 years, while controlling for relevant confounders (age, sex, family income and parental education).

## Methods

### Participants and procedure

**Fig. 2:**
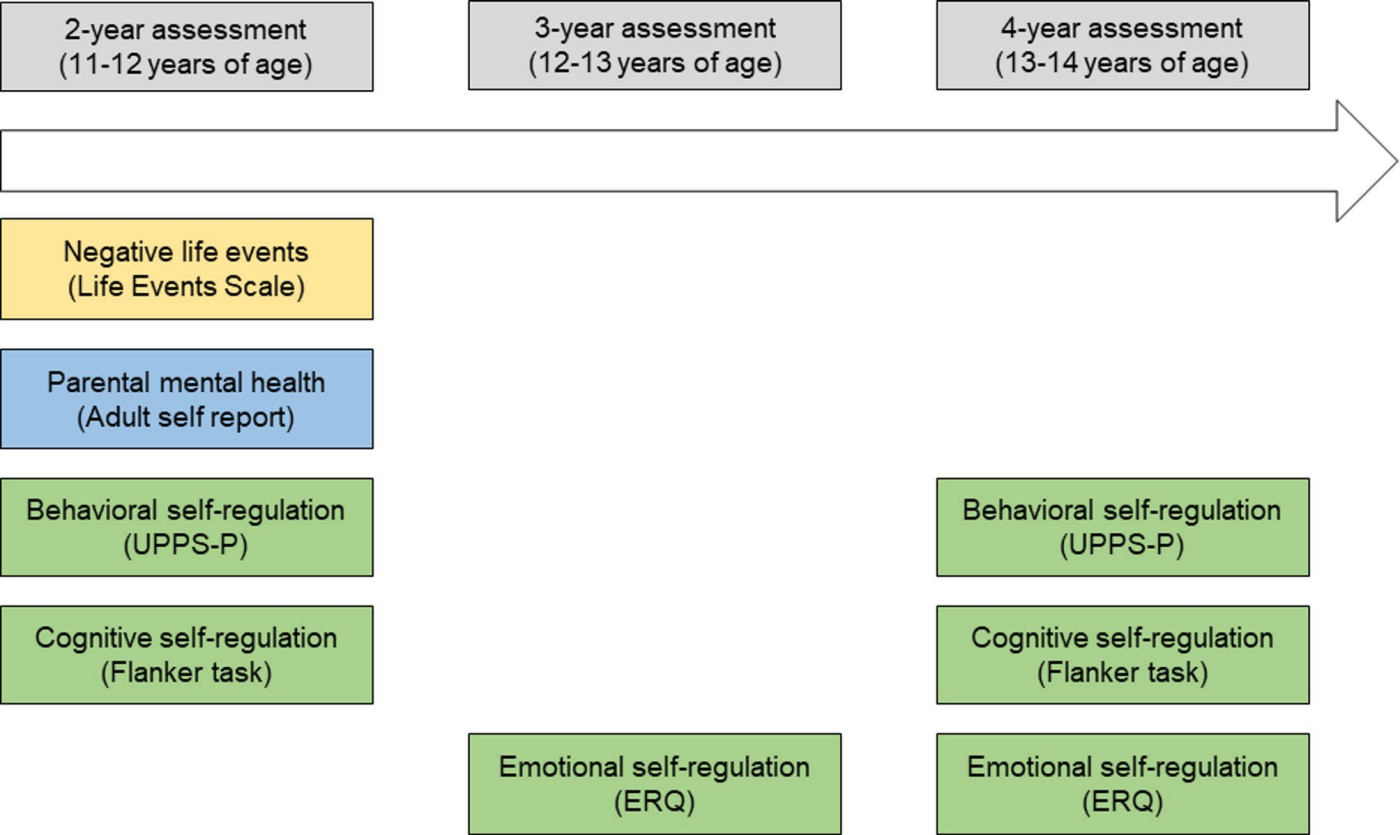
Overview of the constructs used in this study and their assessment points. UPPS-P = Urgency, Premeditation, Perseverance, Sensation Seeking, and Positive Urgency impulsive behavior scale; ERQ = Emotion regulation questionnaire

The data in this study was collected from adolescents and parents as part of the ABCD Study^®^ [29]. This prospective longitudinal study is conducted in the United States of America to investigate the structural and functional brain development and child health. A probability sample of schools in the defined area of the 21 research sites was drawn, and nearly 12,000 eligible children aged 9–10 years and their parents were recruited in the study. The ABCD study aims to follow the children for at least ten years and to examine them regularly every 6 to 12 months [39, 40]. Study procedures were approved by the institutional review boards of the University of California, San Diego (IRB# 160091) and the data collection sites. Written informed consent was obtained from all parents and adolescents. The data used in this study are from 2-, 3- and 4-year assessments when adolescents were 11–12, 12–13, and 13–14 years old, respectively (see Fig. 2). The 1-year assessment was excluded, because the measures used to assess self-regulation (ERQ, Flanker, UPPS-P) and parental mental health problems were not used at this assessment. Data were published as data version 5.1 and collected between September 2016 and January 2022 (10.15154/z563-zd24).

At 4-year assessment, *N* = 4384 adolescents were still available with data on the variables included in this study. Most missing data occurred in the Flanker task at 4-year assessment (*n* = 1287, 29.4%). Like other studies that used data from the ABCD study [95–97], we decided to exclude cases with missing data on the flanker task. For the other variables less than 5% of the data was missing overall. To handle missing data consistently, we also decided for excluding cases with missing data on the other outcome variables. After excluding participants (*N* = 1581) with missing data for a variable of interest (NLEs, parental mental health problems, self-regulation, control variables) at any assessment point, our final analysis sample consisted of *N* = 2803 (see Table S1 and Table S2 for sample characteristics). Adolescents who had to be excluded had parents with lower education (Wilcoxon test: *W* = 2316410, *p* < 0.001) than the participants who were included in the analysis but did not differ in terms of child age, child sex and family income.

### Measures

#### Self-regulation

Self-regulation can be subdivided in three components (i.e. emotional, behavioral and cognitive), which were each tested with a different instrument in the ABCD study. Behavioral and cognitive self-regulation were assessed when adolescents were 11–12 (2-year assessment) and 13–14 (4-year assessment) years old and emotional self-regulation was assessed when adolescents were 12–13 (3-year assessment) and 13–14 (4-year assessment) years old (see also Fig. 2).

To assess *behavioral self-regulation*, we used the short version of the Urgency, Premeditation, Perseverance, Sensation Seeking, and Positive Urgency (UPPS-P) impulsive behavior scale [41] filled in by the adolescents. Five subscales are assessed with 20 items, each rated on a 4-point Likert scale (1 = *Very much like me* to 4 = *Not at all like me*; Whiteside and Lynam [42]). Five factors of impulsive behavior are assessed: *(Negative) Urgency*, *Lack of Perseverance*, *Lack of Premediation/Planning*, *Sensation Seeking* and *Positive Urgency*. We calculated UPPS-P total scores by summing the five subscale scores at each time point (2- and 4-year assessment). The total score ranges between 20 and 80 and higher scores indicate higher levels of impulsivity. UPPS-P showed acceptable internal consistency in other samples (α = 0.76; [43]) and in our sample (α = 0.82) and good convergent and discriminant validity in previous studies [44].

To assess *cognitive self-regulation*, or effortful control, we used the Toolbox Flanker Task^®^ [45]. This task is a variant of the Eriksen Flanker task [46] and assesses attention and cognitive control [45] and consists of 40 trials [11]. In this task, participants should focus on an arrow in the center (stimulus) of the screen that is flanked by other arrows (‘flankers’). In the congruent condition, the flankers point in the same direction as the stimulus, in the incongruent condition, they point in the opposite direction. Participants are asked to press the key that corresponds to the direction of the stimulus [45, 47]. In previous studies, the task showed excellent test-retest reliability (ICC = 0.92) in 3-15-year-olds and adequate convergent validity (*r* = .34) in 8-15-year-olds. The test scores are based on speed and accuracy, with higher scores indicating better performance.

The Emotion Regulation Questionnaire for Children and Adolescents (ERQ; [48, 49]) was used to assess self-reported *emotional self-regulation*. The questionnaire is based on the 10-item original Emotion Regulation Questionnaire by Gross and John [50]. For the ABCD study, three items assessing *cognitive reappraisal* (e.g., “When I want to feel less bad (e.g., sad, angry, worried) about something, I think about something different”) and three items assessing *expressive suppression* (e.g., “I control my feelings by not showing them”) were selected. The items are answered on a five-point scale (1 = *strongly disagree* to 5 = *strongly agree*). Total scores for both scales range between three and 15, and higher scores indicate a greater tendency to use the corresponding emotion regulation strategy. In previous studies, the ERQ has shown adequate convergent validity and good internal consistency (α = 0.83 for cognitive reappraisal and α = 0.75 for expressive suppression; [49]). In our sample, the ERQ showed acceptable internal consistency (α = 0.72 for cognitive reappraisal and α = 0.75 for expressive suppression).

#### Negative life events

The Life Events Scale [51] assesses lifetime and past-year NLEs. For every life event, the adolescents rated whether the event happened (“Did this happen in the past year?”; 1 = *Yes*; 0 = *No*) and how they experienced the event (“Was this a good or bad experience?; 1 = *Mostly good*; 2 = *Mostly bad*; 6 = *Not applicable*; 7 = *Don’t know*). In our analyses, we only included sum scores (i.e. total number) of different lifetime NLEs, operationalized by events that were experienced as *mostly bad* (see Table 1 for the frequency of the 10 most common NLEs in the current sample).

**Table 1:** Frequency of the 10 most common negative life events

Negative life event	*n* (%)
Someone in family died	1540 (54.9)
Family member was seriously injured	830 (29.6)
Lost a close friend	454 (16.2)
Saw crime or accident	408 (14.6)
You got seriously injured	366 (13.1)
Parents separated or divorced	325 (11.6)
You got seriously sick	313 (11.2)
Close friend was seriously sick/injured	305 (10.9)
Family member had drug and/or alcohol problem	247 (8.8)
Family member had mental/ emotional problem	210 (7.5)

All events were experienced “Mostly bad” by the adolescents; multiple answers are possible

#### Parental mental health problems

The Adult Self Report (ASR; Achenbach and Rescorla [52]) was used to screen for parental mental health problems. The 120 items are rated by the parents based on the preceding six months on a 3-point scale (0 = *not true*, 1 = *somewhat or sometimes true*, or 2 = *very true or often true)*. A total problems score can be calculated with a range of 0 to 240. A t-value of 63 or higher is considered clinically relevant and is used in our study to differentiate between parents with and without clinically relevant mental health problems. Internal consistency of the ASR in previous studies (α = 0.91 (Internalizing), α = 0.84 (Externalizing) in de Vries, van de Weijer [53]) and in our sample was excellent (α = 0.94).

#### Control variables

In order to account for characteristics that prior studies found to be associated with self-regulation [54, 55], we controlled for participants’ age at baseline (in years), sex (binary: male/ female), family income (categorical) and parental education (categorical).

### Data analysis

Data analyses were conducted using R Studio 2023.12.1 [56] and IBM SPSS Statistics 29.0 [57]. To examine mean differences between adolescent self-regulation at the two time points, mixed Analyses of Variances (ANOVAs) were used to test main and interaction effects of time (age of 11–12 and 12–13 years for effortful control and impulsivity; age of 12–13 and 13–14 years for expressive suppression and cognitive reappraisal) and group (presence or absence of NLEs or clinically significant parental mental health problems at the age of 11–12 years). The *rstatix* package was used for mixed ANOVAs, and when prerequisites were violated, robust mixed ANOVAs on trimmed means were run using the WRS2 package (*bwtrim* function). Individual-level changes were assessed via the Reliable Change Index (RCI) using the *JTRCI* package, identifying clinically significant differences beyond measurement error [58]. To evaluate the effects of the number of NLEs and severity of parental mental health problems on self-regulation, controlling for self-regulation at 2-year assessment (age 11–12), age, sex, family income, and parental education, multiple linear regression was applied. We performed a bias-corrected and accelerated bootstrapped (BCa-method, *N* = 1,000 repetitions) regression analysis because data were not normally distributed. Effect sizes were classified into small (*r* ≥ .10, η^2^_part_ ≥ 0.01, *R*^*2*^ ≥ 0.01), medium (*r* ≥ .30, η^2^_part_ ≥ 0.06, *R*^*2*^ ≥ 0.09) and large effects (*r* ≥ .50, η^2^_part_ ≥ 0.14, *R*^*2*^ ≥ 0.25) [59].

## Results

### Mean and individual changes in self-regulation and group differences

#### Effortful control (cognitive self-regulation)

There was a significant increase over time in effortful control, *F*(1, 1682) = 736.80, *p* < .001 with η^2^_part_ = 0.22, indicating a large effect. The RCI showed that 84.2% of adolescents did not show a reliable change over the 2-year period. A clinically significant improvement was shown by 10.0% of adolescents and 1.5% showed a decline. A percentage of 4.3 showed a non-reliable improvement. All test statistics can be found in Table S4. No group differences were found between adolescents with and without NLEs and parents with clinically significant mental health problems.

#### Impulsivity (behavioral self-regulation)

Impulsivity significantly increased over time, *F*(1, 1682) = 231.45, *p* < .001 with η^2^_part_ = 0.09 indicating a medium effect. No reliable change in impulsivity over the 2-year period was shown by 68.1% of adolescents. A clinically significant decline was shown by 8.5% and 23.4% showed an increase. Group comparisons revealed significant differences with higher levels of impulsivity in adolescents with NLEs compared to those without NLEs, *F*(1, 414.22) = 10.95, *p* = .001 with η^2^_part_ < 0.01 indicating a very small effect. Moreover, adolescents with parents with clinically significant mental health problems showed higher levels of impulsivity compared to adolescents without, *F*(1, 1758.57) = 4.67, *p* = .031, with η^2^_part_ < 0.01 indicating a very small effect. All test statistics can be found in Table S5.

#### Cognitive reappraisal (emotional self-regulation)

There was no significant change in cognitive reappraisal on the mean level over time, *F*(1, 1682) = 0.18, *p* = .670. The RCI indicated no reliable change in cognitive reappraisal over the 1-year period in 91.2% of adolescents. Furthermore, 5.0% showed a decrease and 3.8% showed a significant increase in their cognitive reappraisal score. There was a significant interaction effect of time and group for cognitive reappraisal, *F*(1, 387.80) = 4.14, *p* = .05, with η^2^_part_ < 0.01 indicating a very small effect. No group differences were found between adolescents with and without NLEs and parents with clinically significant mental health problems. All test statistics can be found in Table S6.

#### Expressive suppression (emotional self-regulation)

Expressive suppression significantly increased over time, *F*(1, 1682) = 209.80, *p* < .001, with η^2^_part_ = 0.07 indicating a medium effect. The RCI showed, that most adolescents (87.9%) did not show a reliable change in the expressive suppression score over the 2-year period. A percentage of 9.4% showed a significant increase and 2.7% showed a significant decrease in the expressive suppression score. Group comparisons revealed significantly higher use of expressive suppression in adolescents who experienced NLEs, *F*(1, 378.98) = 5.02, *p* = .026, with η^2^_part_ = 0.002 indicating a very small effect. No group effect was found for parental mental health problems. All test statistics can be found in Table S7.

### Predictive effect of the number of lifetime NLEs and severity of parental mental health problems on adolescent self-regulation

#### Effortful control (cognitive self-regulation)

The regression model explained 24.6%, *F* = 131.59, *p* < .001 of the variance of effortful control at the age of 13–14 years. Neither NLEs nor parental mental health problems significantly predicted effortful control at the age of 13–14 years, while effortful control at the age of 11–12 years (ß = 0.43, *p* < .001), higher age (ß = 0.04, *p* = 0.031), male sex (ß = − 0.07, *p* < .001), higher family income (ß = 0.13, *p* < .001) and higher parental education (ß = 0.05, *p* = .028) were significant predictors (see Table 2).

#### Impulsivity (behavioral self-regulation)

A total of 29.0% (*F* = 164.70, *p* < .001) of the variance of impulsivity at the age of 13–14 years was explained by the regression model. Besides NLEs (ß = 0.04, *p* = .020) and parental mental health problems (ß = 0.08, *p* < .001), impulsivity at the age of 11–12 years (ß = 0.53, *p* < .001), and female sex (ß = 0.03, *p* = .026) significantly predicted impulsivity at the age of 13–14 years (see Table 2).

#### Cognitive reappraisal (emotional self-regulation)

The regression model explained 8.3% (*F* = 37.19, *p* < .001) of the variance of cognitive reappraisal. NLEs and parental mental health problems were no predictors of cognitive reappraisal. In addition to cognitive reappraisal at the age of 12–13 years (ß = 0.27, *p* < .001), age (ß = 0.05, *p* = .014), male sex (ß = − 0.05, *p* = .011), and parental education (ß = − 0.08, *p* < .001) significantly predicted cognitive reappraisal at the age of 13–14 years (see Table 2).

#### Expressive suppression (emotional self-regulation)

A total of 15.3% (*F* = 73.21, *p* < .001) of the variance of expressive suppression at the age of 13–14 years was explained by the regression model. Parental mental health was no significant predictor. In addition to NLEs (ß = 0.04, *p* = .019), expressive suppression at the age of 12–13 years (ß = 0.38, *p* < .001) and female sex (ß = 0.06, *p* < .001) significantly predicted expressive suppression at the age of 13–14 years (see Table 2).

**Table 2:** Multiple regression analyses for the prediction of self-regulation at the 4-year assessment (*n* = 2803) including NLEs, parental mental health problems and control variables

Variable	Effortful control		Impulsivity		Cognitive reappraisal		Expressive suppression	
	Standardized Regression Coefficient ß^a^ (SE)	*p*-value^a^ [95% CI^a^]	Standardized Regression Coefficient ß^a^ (SE)	*p*-value^a^ [95% CI^a^]	Standardized Regression Coefficient ß^a^ (SE)	*p*-value^a^ [95% CI^a^]	Standardized Regression Coefficient ß^a^ (SE)	*p*-value^a^ [95% CI^a^]
**Adversities**								
Negative life events (11–12 years of age)	0.00 (0.6)	0.891 [-0.12; 0.13]	0.04 (0.06)	0.020 [0.03; 0.24]	0.02 (0.02)	0.299 [-0.02; 0.06]	0.04 (0.02)	0.019 [0.01; 0.09]
Parental mental health problems (11–12 years of age)	0.02 (0.1)	0.183 [-0.01; 0.04]	0.08 (0.01)	< 0.001 [0.04; 0.09]	− 0.03 (0.00)	0.091 [-0.02; 0.00]	0.01 (0.01)	0.647 [-0.01; 0.01]
**Control variables**								
Outcome measure at 11–12 years of age (effortful control and impulsivity) and 12–13 years of age (cognitive reappraisal and expressive suppression)	0.43 (0.2)	< 0.001 [0.39; 0.46]	0.53 (0.02)	< 0.001 [0.49; 0.56]	0.27 (0.02)	< 0.001 [0.22; 0.30]	0.38 (0.02)	< 0.001 [0.34; 0.42]
Age	0.04 (0.23)	0.031 [0.08; 1.03]	− 0.02 (0.24)	0.292 [-0.77; 0.20]	0.05 (0.08)	0.014 [0.03; 0.40]	0.02 (0.09)	0.171 [-0.06; 0.29]
Sex	− 0.07 (0.23)	< 0.001 [-1.44; -0.50]	0.03 (0.25)	0.026 [0.06; 0.98]	− 0.05 (0.08)	0.011 [-0.38; − 0.06]	0.06 (0.09)	< 0.001 [0.14; 0.51]
Family income	0.13 (0.07)	< 0.001 [0.26; 0.55]	0.03 (0.07)	0.100 [-0.02; 0.25]	− 0.00 (0.02)	0.944 [-0.05; 0.05]	− 0.02 (0.03)	0.436 [-0.08; 0.03]
Parental education	0.05 (0.06)	0.028 [0.01; 0.28]	− 0.03 (0.06)	0.133 [-0.21; 0.03]	− 0.08 (0.02)	< 0.001 [-0.11; − 0.04]	− 0.03 (0.02)	0.262 [-0.07; 0.01]
**Test statistics**								
Corrected *R*^2^		0.246		0.290		0.083		0.153
*F* (*p*-value)	131.59 (< 0.001)		164.70 (< 0.001)		37.19 (< 0.001)		73.21 (< 0.001)	

^a^Bootstrapped values

## Discussion

This study examined individual and mean changes in early adolescent self-regulation over two years (behavioral and cognitive self-regulation) and one year (emotional self-regulation) that indicate improvements in some components of self-regulation and decreased skills in others. More specifically, they showed a significant weak increase in cognitive self-regulation and expressive suppression of emotions and a decrease in behavioral self-regulation (i.e., an increase in impulsivity). Group comparisons revealed significant differences with higher levels of impulsivity and higher use of expressive suppression in adolescents with NLEs compared to those without NLEs and higher levels of impulsivity in adolescents with parents with clinically relevant mental health problems compared to those with parents without clinically relevant mental health problems. Moreover, NLEs and parental mental health predicted impulsivity at age 13–14 and NLEs predicted expressive suppression at age 13–14.

While average self-regulation showed slight changes over time, most children remained stable individually, with only a few exhibiting significant increase or decline. In general, early childhood is often considered as a critical developmental period for self-regulation [9], and some studies indicate a relatively stable trajectory of self-regulation from early childhood into adolescence (e.g [60]). Although the observed effects are weak, it is important to note that even within a relatively short period of 1–2 years, meaningful changes in self-regulation can occur. This highlights the dynamic nature of adolescent development and underscores the potential value of early detection and targeted prevention. Additionally, the findings highlight distinct developmental trajectories, indicating groups of children who differ in their self-regulation development [61] impacted by individual risk and protective factors (e.g [62]). Future research needs to focus on exploring these individual pathways and the factors influencing them, ideally over longer time spans and with more than two assessment points to model developmental trajectories.

Consistent with prior research [63, 64] we observed a slight increase in cognitive self-regulation over time. In contrast, behavioral self-regulation declined (reflected by increased impulsivity) similar to patterns found by Littlefield, Stevens [65]. Even small, this effect can be of clinical relevance since they cover 1–2 years of child’s development. The increase in impulsivity may reflect neurodevelopmental imbalances during adolescence, where subcortical regions involved in emotional processes (e.g. amygdala) mature earlier than prefrontal areas responsible for cognitive processes and impulse control [66]. Effortful control, a more voluntary mechanism, is thought to counteract more automatic impulses [67]. Although our data did not examine this and also did not show any association between impulsivity and effortful control scores (see Table S3), previous research suggests that the interaction between effortful control and impulsivity may be a key in understanding the emergence of mental health problems [68]. Future studies could therefore explore not only individual aspects of self-regulation but also how these components interact.

Regarding emotion regulation, our findings align with Gullone, Hughes [69], who observed relatively stable use of cognitive reappraisal in 9- to 15-year-olds over two years. In contrast, Willner, Hoffmann [70] found mostly increasing reappraisal from middle childhood to adolescence in their review, though results were mixed. This discrepancy may stem from our study’s shorter time frame of just one year. While Gullone, Hughes [69] reported a decline in expressive suppression between ages 10 and 14, our data showed a significant increase. Additionally, ERQ scores in our sample were generally lower than those reported by Gullone, Hughes [69]. Herd, King-Casas [71] proposed that emotion regulation tends to improve with age, especially in individuals with lower initial levels. Our results reflect this trend, though variations in baseline suppression remain unexplained. Sex differences in emotion regulation, noted by Nolen-Hoeksema and Aldao [72] and Zimmermann and Iwanski [73] were also evident in our study, underscoring the importance of considering sex in future research. Finally, Aldao and Nolen-Hoeksema [74] emphasized the interplay between adaptive and maladaptive strategies, highlighting the need to examine not just individual approaches but also how they interact.

Overall, our results provide initial evidence of small effects of NLEs and parental mental health problems on adolescent self-regulation. These results can be due to our current methodology (e.g. operationalization and analytical strategy). It might also be that self-regulation may be influenced by other environmental factors, may develop earlier in life, or be largely innate. Moreover, characteristics other than the number of NLEs may influence self-regulation. Different types of NLEs have been linked to distinct neurobiological changes [75] which could affect self-regulation in diverse ways [25, 37, 76]. Furthermore, the effects of NLEs appear to differ depending on the timing and developmental stage at which they occur [77]. The effects of parenting on child self-regulation seem to be stronger the younger the children and decrease over time [78]. This could also apply to the effect of parental mental health problems. In addition to behavioral aspects of parental mental health problems, the possible genetic influence on self-regulation must also be considered, as it appears to be as important as environmental influences [79]. Consistent with this, earlier self-regulation at ages 11–12 predicted levels at 13–14 in our study. Longitudinal designs are crucial to explore how self-regulation develops over time, and which factors predict the development, how preexisting self-regulation skills might shape experiences of NLEs and parental mental health problems, and the potential bidirectional relationships among these factors.

Besides earlier self-regulation, child sex was the strongest predictor of self-regulation at ages 13–14 years. While male sex predicted higher scores in effortful control and cognitive reappraisal, female sex was associated with higher scores in impulsivity and expressive suppression. Brain imaging studies suggest sex differences in brain development during adolescence [80], which are in turn related to different components of self-regulation [81] and may therefore help to explain the effect of sex on self-regulation. There are, for example, findings from brain imaging studies indicating that females may be more vulnerable for impulsive behavior during adolescence [82]. Neural mechanisms underlying processes of emotional self-regulation were also found to differ between adolescent females and males [83]. However, the exact mechanisms are not yet completely understood and more research would be needed to investigate how brain structures that are related to the development of the different components of self-regulation differ between females and males.

Although this study included a community sample, our results also have important practical and clinical implications. Self-regulation is widely recognized as a key factor in the development of mental health problems [31, 32]. Our results indicate that there are small mean changes in self-regulation in adolescents over a period of 1 or 2 years and there were significant effects of NLEs and parental mental health problems on the mean change in impulsivity and of NLEs on the mean change in expressive suppression, indicating that normative developmental changes can be affected by adversities. Since self-regulation serves as a protective factor supporting healthy development, early prevention programs targeting these skills could reduce the risk of mental health issues - both in the general population and especially among high-risk groups, such as children exposed to NLEs or parental mental health problems [31, 32, 84–87]. Furthermore, self-regulation problems during early childhood can trigger a cascade of clinical problems across the lifespan if no action is taken [88–91]. Therefore, promoting self-regulation skills early addresses infant mental health concerns and acts as a prevention strategy for future mental health problems.

This study has several limitations. Most data relied on adolescent self-reports, with the Flankers task as the only cognitive test. While this is more objective, it leads to a further limitation as different assessment methods (e.g. tests vs. questionnaires) for self-regulation show discrepancies and only questionnaires were shown to be related to everyday behavior [92]. Additionally, only the number of different NLEs was recorded without accounting for repeated occurrences of the same event or timing, making it impossible to control for self-regulation levels before the NLEs. However, using the number of NLEs remains a common approach, allowing comparison with prior research. We also did not analyze the impact of specific types of NLEs or those occurring after age 11–12 (2-year assessment), which may influence self-regulation. Our study did not consider genetic or fetal programming factors emphasized in models of transgenerational transmission of self-regulation [93, 94], though we included key environmental influences such as NLEs and parental mental health problems. Moreover, adolescents excluded due to missing data differed in parental education from those included, potentially limiting generalizability and introducing bias, although other sociodemographic factors were similar. Furthermore, the high attrition rate in the ABCD study may also limit the validity of the study. Another limitation was the different assessment time points of the outcome variables (behavioral and cognitive self-regulation were only assessed when adolescents were 11–12 (2-year assessment) and 13–14 (4-year assessment) years old and emotional self-regulation was only assessed when adolescents were 12–13 (3-year assessment) and 13–14 (4-year assessment) years old) which hampered modelling a latent variable at 2 years and comparison of the results between the three components. Moreover, more complex statistical approaches such as structural equation models could lead to a better understanding of dynamic developmental trajectories and their determinants. However, due to limitations in the available data (not all measurements were available at all assessment points, only two assessment points were considered) we opted for ANOVA as a robust and parsimonious model to investigate mean differences in self-regulation measurements between time points to enhance interpretability. Finally, the relatively short developmental period examined is a constraint. Nonetheless, the study comprised multiple assessment points and a longitudinal design. Alongside these limitations, the study has notable strengths. Its longitudinal design includes a large adolescent sample and incorporates data from both adolescents and their parents. Validated measures were used in the study and confounding variables were also assessed.

## Conclusion

In summary, our results suggest small changes in adolescent self-regulation over a period of 1–2 years. These changes were marked by both improved skills and increased difficulties in different components of self-regulation indicating complex development in this sensitive phase. As the period included in this study was quite short, these weak effects might be of relevance to better understand the development of self-regulation in early adolescence and inform targeted prevention. Future studies need to examine longer developmental periods and ideally follow children from infancy to adulthood in a prospective longitudinal design. To better understand the development, adverse experiences and their characteristics such as timing should also be considered. In this way, bidirectional influences between self-regulation and both NLEs and parental mental health problems as well as processes that mediate the effects between these variables can be investigated.

## Supplementary Information

Supplementary Material 1.

## Data Availability

The datasets generated and analyzed during the current study are available in the Adolescent Brain Cognitive Development (ABCD) Study held in the NIMH Data Archive (NDA), https://abcdstudy.org.

## Abbreviations

ASR: Adult self-report
ERQ: Emotion regulation questionnaire
NLE: Negative life events
UPPS-P: Urgency, Premeditation, Perseverance, Sensation Seeking, and Positive Urgency
