# Supplementary material for: The role of negative life events and parental mental health in adolescent self-regulation: insights from the longitudinal ABCD study
Source: Child Adolesc Psychiatry Ment Health. 2025 Dec 1;19:136. doi: 10.1186/s13034-025-00991-5 (PMC12670767; doi:10.1186/s13034-025-00991-5)
Supplement: Supplementary file 1 — Supplementary Material 1. [file 13034_2025_991_MOESM1_ESM.docx]

# Supplement

***Table S1. Sociodemographic variables of included compared to excluded participants***

|  | ***M* (*SD*)** | | Test statistics |
| --- | --- | --- | --- |
|  | Included (*n* = 2803) | Excluded (*n* = 1581) |  |
| Age | 9.52 (0.51) | 9.49 (0.50) | *W* = 2269043 (*p* = .108) |
| Male sex | 52.12% | 52.12% | *W* = 2213674 *(p* = .984) |
| Family income | 7.45 (2.23) | 7.45 (2.20) | *W* = 1777251 (*p* = .898) |
| Parental education | 16.96 (2.55) | 16.65 (2.79) | *W* = 2316410 (*p* = .004) |

|  | **Total sample** | **Group without NLEs vs. with NLEs** | | | | | **Group without vs. with parents with clinically significant mental health problems** | | | | |
| --- | --- | --- | --- | --- | --- | --- | --- | --- | --- | --- | --- |
| **Variable** | **Mean (*SD*)**  (*n* = 2803) | **Mean (*SD*) without NLE**  (*n* = 469) | **Mean (SD) with NLE**  (*n* = 2334) | **Test statistic** | ***p*** | **Effect size** | **Mean (SD) without parents with clinically significant mental health problems**  (*n* = 2726) | **Mean (SD) with parents with clinically significant mental health problems**  (*n* = 77) | **Test statistic** | ***p*** | **Effect size** |
| **Socioeconomic data** |  |  |  |  |  |  |  |  |  |  |  |
| Age at baseline | 9.52 (0.51) | 9.57 (0.50) | 9.51 (0.51) | W = 579033 | .022 | *r* < .1 | 9.52 (0.51) | 9.53 (0.50) | W = 103479 | .808 | *r* < .1 |
| Male sex | 52.1% | 49.3% | 52.7% | W = 566181 | .173 | *r* < .1 | 52.1% | 53.2% | W = 106164 | .841 | *r* < .1 |
| Family income | 7.45 (2.23) | 7.81 (2.06) | 7.38 (2.26) | W = 612661 | < .001 | *r* < .1 | 7.48 (2.22) | 6.24 (2.24) | W = 141168 | < .001 | *r* < .1 |
| Parent education | 16.96 (2.55) | 17.38 (2.23) | 16.88 (2.61) | W = 610023 | < .001 | *r* < .1 | 16.98 (2.57) | 16.21 (1.94) | W = 132605 | < .001 | *r* < .1 |
| **Self-regulation** |  |  |  |  |  |  |  |  |  |  |  |
| ***Effortful control (Flanker)*** |  |  |  |  |  |  |  |  |  |  |  |
| 11-12 years of age | 100.65 (7.28) | 101.19 (7.29) | 100.54 (7.27) | W = 577290 | .06 | *r* < .1 | 100.66 (7.29) | 100.26 (6.73) | W = 111356 | .36 | *r* < .1 |
| 13-14 years of age | 104.59 (7.13) | 105.13 (6.81) | 104.49 (7.19) | W = 570711 | .14 | *r* < .1 | 104.62 (7.10) | 103.77 (8.14) | W = 111117 | .378 | *r* < .1 |
| ***Impulsivity (UPPS-P)*** |  |  |  |  |  |  |  |  |  |  |  |
| 11-12 years of age | 39.16 (7.72) | 38.16 (7.35) | 39.36 (7.78) | W = 502039 | .005 | *r* < .1 | 39.14 (7.68) | 39.79 (8.90) | W = 100710 | .545 | *r* < .1 |
| 13-14 years of age | 41.43 (7.68) | 40.29 (6.93) | 41.66 (7.81) | W = 491320 | <.001 | *r* < .1 | 41.37 (7.67) | 43.42 (7.84) | W = 87331 | .012 | *r* < .1 |
| ***Cognitive reappraisal (ERQ)*** |  |  |  |  |  |  |  |  |  |  |  |
| 12-13 years of age | 10.07 (2.35) | 10.09 (2.27) | 10.06 (2.36) | W = 552866 | 0.726 | *r* < .1 | 10.08 (2.35) | 9.81 (2.21) | W = 111498 | .345 | *r* < .1 |
| 14-15 years of age | 10.10 (2.28) | 9.96 (2.16) | 10.13 (2.30) | W = 518983 | 0.073 | *r* < .1 | 10.11 (2.28) | 9.88 (2.34) | W = 108183 | .641 | *r* < .1 |
| ***Expressive suppression (ERQ)*** |  |  |  |  |  |  |  |  |  |  |  |
| 12-13 years of age | 9.12 (2.55) | 9.02 (2.31) | 9.14 (2.60) | W = 531653 | 0.324 | *r* < .1 | 9.12 (2.56) | 9.27 (2.41) | W = 99708 | .451 | *r* < .1 |
| 14-15 years of age | 9.89 (2.58) | 9.61 (2.49) | 9.94 (2.60) | W = 504111 | .007 | *r* < .1 | 9.89 (2.58) | 9.87 (2.68) | W = 106186 | .859 | *r* < .1 |
| **Adversities** |  |  |  |  |  |  |  |  |  |  |  |
| Number of NLEs | 2.38 (2.16) | 0 (0) | 2.86 (2.06) | W = 0 | <.001 | *r* = .65 | 2.36 (2.14) | 3.06 (2.77) | W = 90112 | .031 | *r* < .1 |
| Parental psychopathology (ASR at 12-13 years of age) | 43.02 (9.80) | 42.26 (9.61) | 43.17 (9.84) | W = 520140 | .089 | *r* < .1 | 42.36 (9.09) | 66.26 (3.97) | W = 0 | <.001 | *r* = 0.28 |

***Table S2.*** *Sample characteristics and mean differences between groups.*

# *Table S3. Pearson correlations r (p-value) of control variables, NLE, parental psychopathology and self-regulation (n = 2803).*

| Variable | 1 | 2 | 3 | 4 | 5 | 6 | 7 | 8 | 9 | 10 | 11 | 12 | 13 |
| --- | --- | --- | --- | --- | --- | --- | --- | --- | --- | --- | --- | --- | --- |
| 1. Age |  |  |  |  |  |  |  |  |  |  |  |  |  |
| 2. Sex | -.01  [-.05, .02] |  |  |  |  |  |  |  |  |  |  |  |  |
| 3. Family income | .06**  [.03, .10] | -.01  [-.04, .03] |  |  |  |  |  |  |  |  |  |  |  |
| 4. Parental education | .02  [-.02, .06] | .02  [-.02, .05] | .62**  [.59, .64] |  |  |  |  |  |  |  |  |  |  |
| 5. Effortful control (Flanker score at 11-12 years of age) | .07**  [.03, .10] | [-.05, .02] | .16**  [.12, .20] | .17**  [.13, .20] |  |  |  |  |  |  |  |  |  |
| 6. Effortful control (Flanker score at 13-14 years of age) | .07**  [.04, .11] | -.08**  [-.11, -.04] | .23**  [.19, .26] | .20**  [.16, .23] | .46**  [.43, .49] |  |  |  |  |  |  |  |  |
| 7. Impulsivity (UPPS-P score at 11-12 years of age) | .03  [-.01, .07] | -.14**  [-.18, -.11] | -.05*  [-.08, -.01] | -.03  [-.07, .01] | .01  [-.03, .05] | .00  [-.03, .04] |  |  |  |  |  |  |  |
| 8. Impulsivity (UPPS-P score at 13-14 years of age) | -.01  [-.04, .03] | -.04*  [-.08, -.00] | -.02  [-.06, .01] | -.03  [-.07, .00] | -.00  [-.04, .03] | -.00  [-.04, .03] | .53**  [.50, .56] |  |  |  |  |  |  |
| 9. Cognitive reappraisal (ERQ score at 12-13 years of age) | .01  [-.03, .05] | .05*  [.01, .08] | .01  [-.03, .05] | -.01  [-.05, .03] | .01  [-.03, .05] | .02  [-.02, .06] | -.02  [-.06, .01] | -.02  [-.06, .01] |  |  |  |  |  |
| 10. Cognitive reappraisal (ERQ score at 13-14 years of age) | .05*  [.01, .08] | -.04*  [-.07, -.00] | -.05*  [-.08, -.01] | -.09**  [-.12, -.05] | -.01  [-.05, .03] | .02  [-.02, .05] | -.05**  [-.09, -.02] | -.08**  [-.11, -.04] | .27**  [.23, .30] |  |  |  |  |
| 11. Expressive suppression (ERQ score at 12-13 years of age) | .03  [-.01, .07] | -.01  [-.05, .03] | -.08**  [-.11, -.04] | -.07**  [-.11, -.03] | -.02  [-.05, .02] | .00  [-.04, .04] | .15**  [.11, .19] | .14**  [.10, .17] | .28**  [.24, .31] | .05**  [.02, .09] |  |  |  |
| 12. Expressive suppression (ERQ score at 13-14 years of age) | .03  [-.01, .07] | .06**  [.02, .09] | -.07**  [-.11, -.03] | -.07**  [-.11, -.03] | -.02  [-.06, .01] | -.00  [-.04, .04] | .10**  [.07, .14] | .19**  [.16, .23] | .09**  [.05, .13] | .19**  [.15, .22] | .38**  [.35, .41] |  |  |
| 13. Number of NLEs | -.05**  [-.09, -.02] | -.01  [-.05, .03] | -.20**  [-.24, -.17] | -.17**  [-.21, -.13] | -.05**  [-.09, -.02] | -.05**  [-.09, -.02] | .18**  [.15, .22] | .14**  [.11, .18] | .00  [-.03, .04] | .03  [-.01, .07] | .09**  [.05, .12] | .08**  [.04, .12] |  |
| 14. Parental psychopathology (ASR at 12-13 years of age) | -.04  [-.07, .00] | .00  [-.04, .04] | -.08**  [-.12, -.05] | -.04*  [-.08, -.00] | .02  [-.01, .06] | .02  [-.02, .06] | .08**  [.04, .11] | .12**  [.08, .16] | .00  [-.03, .04] | -.03  [-.07, .01] | .04  [-.00, .07] | .03  [-.01, .07] | .11**  [.07, .15] |

***Note***. UPPS-P = Urgency, Premeditation, Perseverance, Sensation Seeking, and Positive Urgency impulsive behavior scale; ERQ = Emotion regulation questionnaire; NLEs = Negative life events ASR = Adult self-report.

***Table S4.*** Within and between group effects for effortful control for the whole sample, comparing groups without vs. with NLEs and comparing groups without vs. with parents with clinically significant mental health problems.

| **Whole sample** | | | | | **Experience of negative life events** | | | | | **Clinically significant parental mental health problems** | | | | |
| --- | --- | --- | --- | --- | --- | --- | --- | --- | --- | --- | --- | --- | --- | --- |
|  | **df** | **F** | **p-value** | **η^2^_part_** |  | **df** | **F** | **p-value** | **η^2^_part_** |  | **df** | **F** | **p-value** | **η^2^_part_** |
|  |  |  |  |  | **Between-effects** |  |  |  |  | **Between-effects** |  |  |  |  |
|  |  |  |  |  | Group | 1 | 3.81 | .052 | < .01 | Group | 1 | 1.64 | .20 | < .01 |
|  |  |  |  |  | Error term | 396.14 |  |  |  | Error term | 1734.15 |  |  |  |
| **Within effect** |  |  |  |  | **Within effect** |  |  |  |  | **Within effect** |  |  |  |  |
| Time | 1 | 736.80 | < .001 | .22 | Time | 1 | 400.27 | < .001 | .14 | Time | 1 | 67.83 | < .001 | .03 |
|  |  |  |  |  | Interaction | 1 | 0.40 | .530 | < .01 | Interaction | 1 | 0.06 | .799 | < .01 |
| Error term | 1682 |  |  |  | Error term | 392.58 |  |  |  | Error term | 1719.03 |  |  |  |

***Table S5.*** Within and between group effects for impulsivity for the whole sample, comparing groups without vs. with NLEs and comparing groups without vs. with parents with clinically significant mental health problems.

| **Whole sample** | | | | | **Experience of negative life events** | | | | | **Clinically significant parental mental health problems** | | | | |
| --- | --- | --- | --- | --- | --- | --- | --- | --- | --- | --- | --- | --- | --- | --- |
|  | **df** | **F** | **p-value** | **η^2^_part_** |  | **df** | **F** | **p-value** | **η^2^_part_** |  | **df** | **F** | **p-value** | **η^2^_part_** |
|  |  |  |  |  | **Between-effects** |  |  |  |  | **Between-effects** |  |  |  |  |
|  |  |  |  |  | Group | 1 | 10.95 | .001 | < .01 | Group | 1 | 4.67 | .030 | < .01 |
|  |  |  |  |  | Error term | 414.21 |  |  |  | Error term | 1758.57 |  |  |  |
| **Within effect** |  |  |  |  | **Within effect** |  |  |  |  | **Within effect** |  |  |  |  |
| Time | 1 | 231.45 | < .001 | .09 | Time | 1 | 138.23 | < .001 | .05 | Time | 1 | 35.54 | < .001 | .02 |
|  |  |  |  |  | Interaction | 1 | 0.31 | .577 | < .01 | Interaction | 1 | 2.65 | .104 | < .01 |
| Error term | 1682 |  |  |  | Error term | 416.85 |  |  |  | Error term | 1707.09 |  |  |  |

***Table S6.*** Within and between group effects for cognitive reappraisal for the whole sample, comparing groups without vs. with NLEs and comparing groups without vs. with parents with clinically significant mental health problems.

| **Whole sample** | | | | | **Experience of negative life events** | | | | | **Clinically significant parental mental health problems** | | | | |
| --- | --- | --- | --- | --- | --- | --- | --- | --- | --- | --- | --- | --- | --- | --- |
|  | **df** | **F** | **p-value** | **η^2^_part_** |  | **df** | **F** | **p-value** | **η^2^_part_** |  | **df** | **F** | **p-value** | **η^2^_part_** |
|  |  |  |  |  | **Between-effects** |  |  |  |  | **Between-effects** |  |  |  |  |
|  |  |  |  |  | Group | 1 | 0.42 | .518 | < .01 | Group | 1 | 0.54 |  | < .01 |
|  |  |  |  |  | Error term | 394.07 |  |  |  | Error term | 1735.25 |  | .464 |  |
| **Within effect** |  |  |  |  | **Within effect** |  |  |  |  | **Within effect** |  |  |  |  |
| Time | 1 | 0.181 | .517 | < .01 | Time | 1 | 2.77 | .097 | < .01 | Time | 1 | 0.23 | .631 | < .01 |
|  |  |  |  |  | Interaction | 1 | 4.14 | .043 | < .01 | Interaction | 1 | 0.42 | .516 | < .01 |
| Error term | 1682 |  |  |  | Error term | 387.80 |  |  |  | Error term | 1718.54 |  |  |  |

***Table S7.*** Within and between group effects for expressive suppression for the whole sample, comparing groups without vs. with NLEs and comparing groups without vs. with parents with clinically significant mental health problems.

| **Whole sample** | | | | | **Experience of negative life events** | | | | | **Clinically significant parental mental health problems** | | | | |
| --- | --- | --- | --- | --- | --- | --- | --- | --- | --- | --- | --- | --- | --- | --- |
|  | **df** | **F** | **p-value** | **η^2^_part_** |  | **df** | **F** | **p-value** | **η^2^_part_** |  | **df** | **F** | **p-value** | **η^2^_part_** |
|  |  |  |  |  | **Between-effects** |  |  |  |  | **Between-effects** |  |  |  |  |
|  |  |  |  |  | Group | 1 | 5.02 | .026 | < .01 | Group | 1 | 0.11 | .740 | < .01 |
|  |  |  |  |  | Error term | 378.98 |  |  |  | Error term | 1704.76 |  |  |  |
| **Within effect** |  |  |  |  | **Within effect** |  |  |  |  | **Within effect** |  |  |  |  |
| Time | 1 | 209.80 | < .001 | .07 | Time | 1 | 91.82 | < .001 | .03 | Time | 1 | 12.41 | < .001 | < .01 |
|  |  |  |  |  | Interaction | 1 | 2.82 | .094 | < .01 | Interaction | 1 | 0.76 | .384 | < .01 |
| Error term | 1682 |  |  |  | Error term | 391.65 |  |  |  | Error term | 1712.59 |  |  |  |
